# Eye Movements During Everyday Behavior Predict Personality Traits

**DOI:** 10.3389/fnhum.2018.00105

**Published:** 2018-04-13

**Authors:** Sabrina Hoppe, Tobias Loetscher, Stephanie A. Morey, Andreas Bulling

**Affiliations:** ^1^Machine Learning and Robotics Lab, University of Stuttgart, Stuttgart, Germany; ^2^School of Psychology, University of South Australia, Adelaide, SA, Australia; ^3^School of Psychology, Flinders University, Adelaide, SA, Australia; ^4^Perceptual User Interfaces Group, Max Planck Institute for Informatics, Saarbrücken, Germany

**Keywords:** eye tracking, real world, personality, machine learning, gaze behavior, eye-based user modeling

## Abstract

Besides allowing us to perceive our surroundings, eye movements are also a window into our mind and a rich source of information on who we are, how we feel, and what we do. Here we show that eye movements during an everyday task predict aspects of our personality. We tracked eye movements of 42 participants while they ran an errand on a university campus and subsequently assessed their personality traits using well-established questionnaires. Using a state-of-the-art machine learning method and a rich set of features encoding different eye movement characteristics, we were able to reliably predict four of the Big Five personality traits (neuroticism, extraversion, agreeableness, conscientiousness) as well as perceptual curiosity only from eye movements. Further analysis revealed new relations between previously neglected eye movement characteristics and personality. Our findings demonstrate a considerable influence of personality on everyday eye movement control, thereby complementing earlier studies in laboratory settings. Improving automatic recognition and interpretation of human social signals is an important endeavor, enabling innovative design of human–computer systems capable of sensing spontaneous natural user behavior to facilitate efficient interaction and personalization.

Eye movements facilitate efficient sampling of visual information from the world around us. For example, in everyday social interactions, we often understand, predict, and explain the behavior and emotional states of others by how their eyes move (Emery, 2000). The exact mechanisms by which eye movement is controlled, and the range of factors that can influence it, are subject to intense research (Wolfe, 1994; Martinez-Conde et al., 2004; Foulsham et al., 2011; Rucci and Victor, 2015). Understanding the types of information eye movements convey is of current interest to a range of fields, from psychology and the social sciences to computer science (Henderson et al., 2013; Bulling et al., 2011; Bulling and Zander, 2014; Bixler and D'Mello, 2015; Steil and Bulling, 2015). One emerging body of research suggests that the way in which we move our eyes is modulated by who we are—by our personality (Isaacowitz, 2005; Rauthmann et al., 2012; Risko et al., 2012; Baranes et al., 2015; Hoppe et al., 2015).

Personality traits characterize an individual's patterns of behavior, thinking, and feeling (Kazdin, 2000). Studies reporting relationships between personality traits and eye movements suggest that people with similar traits tend to move their eyes in similar ways. Optimists, for example, spend less time inspecting negative emotional stimuli (e.g., skin cancer images) than pessimists (Isaacowitz, 2005). Individuals high in openness spend a longer time fixating and dwelling on locations when watching abstract animations (Rauthmann et al., 2012), and perceptually curious individuals inspect more of the regions in a naturalistic scene (Risko et al., 2012). But pioneering studies on the association between personality and eye movements share two methodological limitations.

First, these early studies typically either investigated the link between gaze and personality descriptively (e.g., using correlation, Risko et al., 2012; Rauthmann et al., 2012) or predicted single gaze characteristics, such as the number of fixations (Isaacowitz, 2005; Risko et al., 2012; Rauthmann et al., 2012), from personality scores. For practical applications, however, the more relevant question is whether, in turn, eye movements can be used to predict personality traits. Intriguingly, machine learning techniques provide a way of answering this question without the need to make a-priori hypotheses about the importance of individual gaze characteristics. Instead, the most informative characteristics can be automatically determined from a potentially large and diverse set of eye movement characteristics and patterns; thereby also uncovering previously unknown links between personality and gaze. The potential of machine learning for predicting behavior, cognitive states and personality has been highlighted in a few studies (Henderson et al., 2013; Bulling and Zander, 2014; Bixler and D'Mello, 2015; Hoppe et al., 2015). A recent laboratory study, for example, successfully predicted people's epistemic curiosity about answers to trivia questions from oculomotor behavior (Baranes et al., 2015).

The second limitation of earlier studies is their restriction to laboratory conditions – an approach that has been criticized because it may not lead to valid theories of human behavior in natural settings (Kingstone et al., 2003, 2008). In most studies, carefully selected stimuli – such as images, animations, or trivia questions – were presented to participants for defined durations on a computer screen, and participants' eye movements were then related to the personality traits under investigation (Isaacowitz, 2005; Rauthmann et al., 2012; Risko et al., 2012; Baranes et al., 2015). However, principles guiding the eyes when looking at computer screens and when engaging in dynamic real-world behavior differ significantly (Foulsham et al., 2011; Tatler et al., 2011; Tatler, 2014). Compelling evidence for such differences is provided in a study which tracked eye movements of participants when they were exploring different real world environments and when watching videos of these environments (Marius't Hart et al., 2009). The distribution of eye movements obtained in the laboratory only predicted the gaze distribution in the laboratory with around 60% accuracy—indicating significant differences in eye movements between laboratory and real world situations (Foulsham et al., 2011). It therefore remains unclear whether these personality traits found to be related to eye movements in the laboratory (Isaacowitz, 2005; Rauthmann et al., 2012; Risko et al., 2012; Baranes et al., 2015) generalize to real-world behaviors. If so, then links between eye movements and personality have important ramifications for the emerging fields of social signal processing, social robotics, and eye-based user modeling. These interdisciplinary fields—at the intersection of computer science, social science, and psychology—focus on the development of systems that can sense, model, and understand everyday human social signals (Vinciarelli et al., 2009; Wagner et al., 2011; Vinciarelli and Pentland, 2015) and that exhibit human-like behavior, including personality (Fong et al., 2003). Ultimately, such socially-aware computers have the potential to offer interactive capabilities that closely resemble natural human-human interactions.

In the present work we demonstrate, for the first time, that the visual behavior of individuals engaged in an everyday task can predict four of the Big Five personality traits (McCrae and Costa, 2010), along with perceptual curiosity (Collins et al., 2004). To this end, we develop and study a large set of features that describe various characteristics of everyday visual behavior. This approach goes beyond existing analyses of individual features and provides a principled demonstration of the link between eye movement and personality. Our findings not only validate the role of personality in explaining eye movement behavior in daily life, they also reveal new eye movement characteristics as predictors of personality traits.

## 1. Methods

Fifty students and staff of Flinders University participated in the study: 42 females and eight males, with a mean age of 21.9 years (SD 5.5). The convenience sample was recruited through an advertisement on the School of Psychology's online participation management system and the sample size was based on Risko et al. (2012). Written informed consent was obtained from all participants and participant received AUD15 for taking part in the study. Ethic approval was obtained from the Human Research Ethics Committee at Flinders University and the study was conducted in accordance with the Declaration of Helsinki.

### 1.1. Apparatus

Binocular gaze data were tracked using a state-of-the-art head-mounted video-based eye tracker from SensorMotoric Instruments (SMI) at 60Hz. The tracker has a reported gaze estimation accuracy of 0.5° and precision of 0.1°. The tracker recorded gaze data, along with a high-resolution scene video on a mobile phone that was carried in a cross-body bag.

### 1.2. Questionnaires

Personality traits were assessed using three established self-report questionnaires: 1) The NEO Five-Factor Inventory (NEO-FFI-3) comprising 60 questions assessing neuroticism, extraversion, openness, agreeableness, and conscientiousness (McCrae and Costa, 2010); 2) Perceptual Curiosity, a 16-item questionnaire assessing a person's interest in novel perceptual stimulation and visual-sensory inspection (Collins et al., 2004); and 3) the Curiosity and Exploration Inventory (CEI-II), a 10-item questionnaire assessing trait curiosity (Kashdan et al., 2009).

### 1.3. Procedure

Upon arrival in the laboratory, participants were introduced to the study and fitted with the eye tracker. The tracker was first calibrated using a standard 3-point calibration routine. Participants were then given AUD5 and instructed to walk around campus for approximately 10 min and to purchase any items of their choice (such as a drink or confectionary) from a campus shop of their choice. Upon return, the tracking was stopped and the glasses were removed. Participants were then asked to fill in the personality and curiosity questionnaires.

## 2. Data processing

The data from one participant were lost due to technical problems with the eye tracking equipment. Any sample where the pupil could not be detected, or the gaze direction was estimated to be beyond 150% of its range, was marked as erroneous. Six participants with more than 50% erroneous samples in their recording were excluded from further analysis; one other participant was excluded because gaze direction was estimated to be constant for 38% of samples. For the remaining 42 participants an average of 12.51 minutes (*SD* = 2.71) of eye tracking data were collected, with an average track loss of 19.58% (*SD* = 0.12). The recording included an average 2.36 minutes inside the shop (*SD* = 1.70).

We independently binned personality scores for each trait into three score ranges (low, medium, and high). The binning was performed in a data-driven fashion so that approximately one third of the participants were assigned to each score range. The middle bin's boundaries were defined as the score percentile at 1/3 and 2/3 respectively. Because personality scores approximately follow a Gaussian distribution, the range of medium scores was smaller than the range for the two extreme classes. Table 2 in the appendix lists all resulting boundaries between score ranges.

Both data and source code are publicly available on GitHub^1^.

### 2.1. Feature extraction

Following best practices in eye-based user modeling (Bulling et al., 2011), the time series of gaze data was processed using a sliding window approach to make the data independent of the individual duration of the recording while not blurring out gaze characteristics due to averaging effects. That is, only data from a time window of a certain length were considered at one time. Different window sizes were evaluated during our training routine (see below for details). The window was slid over the entire recording such that all subsequent windows had an overlap of 50%. Time windows that had more than 50% erroneous samples (i.e., where the pupil could not be detected or the gaze direction was estimated to be beyond 150% of its range), less than 2 non-erroneous samples, or not a single detected fixation or saccade, were discarded. For each resulting time window, a vector of 207 features was extracted (see the Appendix for a list of all features). These features include:

1. Statistics over raw gaze data: These were introduced in Baranes et al. (2015) for the detection of epistemic curiosity under laboratory conditions. Many of the features were specific to the user interface used, for instance the distance of the participant's gaze from a box in the interface but others such as minimum, mean and maximum of gaze x or y coordinates were adopted to our setting.
2. Heatmaps of raw gaze data have been linked to curiosity in a study on a static scene viewing task (Risko et al., 2012). Analogously, an 8 by 8 heatmap of gaze points has been extracted here. Over time a heatmap cell corresponds to different places in the world due to head and body motion. Since some gaze points were extrapolated to positions quite far from the actual scene video, gaze points were only used if they fell within the intervals spanning 95% of the data in both horizontal and vertical direction. The heatmap cells were enumerated from 0 in the top left corner, through 7 in the top right corner, to 63 in the bottom right corner.
3. Statistics over fixations, saccades and blinks have frequently been used in eye tracking studies (Bulling et al., 2011; Rauthmann et al., 2012; Risko et al., 2012). Fixations were detected using a dispersion-threshold algorithm with a threshold of 2.5% of the tracking range width (5) with an additional threshold on the minimum duration of 100ms. All movements between two fixations were inspected as candidate saccades and were accepted if they did not exceed a maximum duration of 500ms and had a peak velocity of at least 200% of the tracking range per second. Both fixations and saccades with more than 50% erroneous samples were discarded. Additionally, the eye tracking software provided information on blinks and pupil diameter. From all events (i.e., fixations, saccades, and blinks), a number of statistics was computed such as the mean duration of fixations and the direction of saccades. A full list of these features can be found in the Appendix.
  Note that “fixations” of up to 500ms are likely to include smooth pursuits that we did not consider separately since robust pursuit detection is still an open research question even for controlled laboratory settings (Hoppe and Bulling, 2016).
4. Information on the temporal course of saccades and fixations has previously been encoded in so-called *n*-gram features for eye-based user modeling (Bulling et al., 2011). *n*-grams describe a series of gaze events, e.g., saccades with different amplitudes (large or small) and directions binned into 8 possible directions (e.g., [“long saccade up,” “short fixation,” “short saccade up”] for *n* = 3). Finally, a histogram of *n*-grams was computed by counting how often each *n*-gram, i.e., each possible combination of saccades and fixations, occurred. For each n between 1 and 4, the following features were extracted from the histogram: number of different *n*-grams (i.e., number of non-zero entries in the histogram), maximum/minimum/mean/variance of the histogram entries and the most/least frequent n-gram.

For each personality trait, a separate random forest classifier (Breiman, 2001) consisting of 100 decision trees was trained on these features to predict one of the three personality score ranges (low, medium, high) using scikit-learn (Pedregosa et al., 2011). Each decision tree resembles a tree-shaped flow-chart of decisions, where we set the maximum depth of each tree to 5 and allowed up to 15 features to be considered per decision. Before each training procedure, a standard scaler was fit to the training data and applied to both training and test samples to ensure a mean of zero and a standard deviation of one for each feature.

We had no a priori hypothesis concerning which window size for the sliding-window approach would be most effective, or which particular features would be useful. We therefore chose an automatic approach named *nested cross validation* to optimize the open parameters during training, i.e., window size and feature selection. In a nutshell, a nested cross validation cycles through sets of participants: one training set, one validation set, and one test set. For instance, in the first iteration, participants 1-32 might be used for training, participants 33–37 for validation, and participants 38–42 for testing. In the second iteration, participants 5–37 might be used for training, then participants 10–42 and so on. In all iterations, several classifiers based on different window sizes and subsets of features were trained on the training set and evaluated on the validation set. The best performing window size and subset of features was chosen based on the performance on the validation set. A classifier was then trained on the union of training and validation set and tested on the test set to generate the final performance scores reported here. It is important to select parameters based on performance on the validation set and then re-train and evaluate on another test set, because with this scheme, the parameters were never directly optimized for the final evaluation. Therefore, cross validation effectively mitigates the risk of overfitting—the algorithm is forced to generalize to unseen data.

### 2.2. Classifier evaluation

Classifier performance was evaluated in terms of average F1 score across the three score ranges. The F1 score for a particular range R is defined as the harmonic mean of *precision* (the probability that the true personality score range for a random person out of those for which R was predicted is indeed R) and *recall* (the probability that R will be predicted for a randomly chosen participant whose true personality score is within R). Since the training procedure for random forest classifiers is inherently non-deterministic, we went through the whole nested cross-validation scheme 100 times with different initial random states.

We compared our classifier against several random baselines to determine how likely our classification success was according to simpler or trivial classifiers:

1. Theoretical chance level: if all predictions were made uniformly at random and all score ranges are equally likely, the resulting F1 score for three balanced classes should be 0.33. Slight deviations from these assumptions, e.g., unbalanced classes, could in practice lead to different results. Thus, we implemented a simple classifier that randomly sampled one of the three score ranges for each person from a uniform distribution.
2. Predicting the most frequent score range: For this evaluation, the training and test set were built in an identical manner to the actual training process, but instead of fitting a classifier, the most frequent score range on the training set was determined and then predicted for every person in the test set. Note that this might be slightly different from the theoretical 33% because the splits into training and test set might distort the label frequencies.
3. The label permutation test (Ojala and Garriga, 2010) was proposed to determine the level of performance after any relation between features and score ranges was obfuscated, i.e., the training data was artificially shuffled such that the relation between gaze and personality was lost. If this classifier is able to perform above a theoretical chance level it might for instance have picked up class frequencies. Thus, it can serve as a test of how much actual information from the gaze features was learned by our original classifier (Bode et al., 2012).

Each of these baselines was computed 100 times, so a set of 100 F1 scores per baseline was obtained and compared to those of our classifier.

## 3. Results

**Figure 1:**
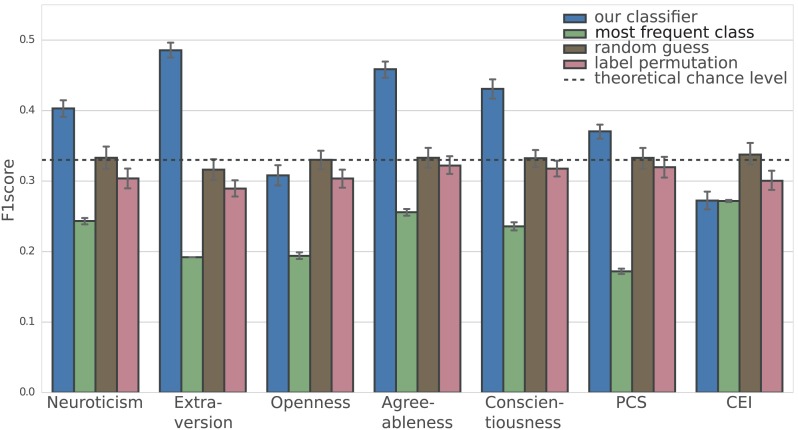
Mean F1 scores of 100 instances of our classifier and three baselines per trait. The whiskers indicate the 95% confidence interval around the mean, computed by bootstrapping with 1,000 iterations on the set of 100 F1 scores for each trait. All results were obtained using a cross-validation scheme such that only predictions for unseen participants were used for evaluation. The dashed line shows the theoretical chance level for a classifier that randomly picks one personality score range for each participant, independent of gaze.

Figure 1 shows the mean F1 score for our classifier as well as for all baselines for each trait. As can be seen from the figure, our classifier performs well above chance (that is, confidence intervals do not overlap with any of the baseline performances) for neuroticism (40.3%), extraversion (48.6%), agreeableness (45.9%), conscientiousness (43.1%), and perceptual curiosity (PCS, 37.1%). For openness (30.8%) and the Curiosity and Exploration Inventory (CEI, 27.2%) our classifier performs below chance level.

In the above evaluation, all recorded data were used irrespective of participants' context: that is, regardless of whether they were on their way to the shop, or inside the shop. To evaluate the reliability of classifiers within and across different parts of the recording, times at which people entered and left the shop were manually annotated based on the recorded scene video. We then compared their predictions across different subsets of the data: (1) independent of the participant's activity (two halves of the recording: *split halves*); (2) within one activity (the way to the shop vs. the way back to the laboratory: *way I vs. II*); and (3) across activities (navigation on the way vs. shopping inside: *shop vs. way*). For each comparison, we used the 100 classifiers trained for the first part of the paper and reconstructed the predictions for single time windows (i.e., the predictions before majority voting). Majority voting was performed over time windows from the context in question only, such as from time windows when the participant was inside the shop. As each classifier had been trained and evaluated 100 times, this led to 100 pairs of predictions for each comparison. Reliability was then evaluated by the average correlation between these pairs of predictions after correction for the skewness of the sampling distribution of correlation coefficients, using the Fisher transformation (Fisher, 1915). The resulting Pearson product-moment correlation coefficients are shown in Table 1. The coefficients ranged from 0.39 to 0.83, indicating a moderate to strong correlation between these different real-world contexts.

**Table 1:** Pearson product-moment correlation coefficients of predictions obtained from different parts of the recording: in the first half vs. the second half (*split halves*), on the way to the shop vs. on the way back to the laboratory (*way I vs. II*) and inside the shop vs. outside the shop (*shop vs. way*).

	**half I vs. half II**	**way I vs. way II**	**shop vs. ways**
Neuroticism	0.77	0.75	0.63
Extraversion	0.83	0.75	0.61
Openness	0.64	0.60	0.39
Agreeableness	0.63	0.56	0.44
Conscientiousness	0.69	0.72	0.43
Perceptual Curiosity	0.68	0.65	0.46
Curiosity and Exploration	0.68	0.65	0.44

To investigate in more detail how eye movement characteristics are linked to individual personality traits, we further calculated the relative importance of all features from the random forest classifier as suggested in Breiman (2001). A random forest classifier comprises several decision trees. The importance of a feature in the random forest is defined as its average importance across all the component decision trees. Within a single decision tree, a feature's importance is defined via all decisions that are made based on that feature: the greater the number of decisions made, the smaller the mean classification error and the more data is passed through these decisions in the tree structure, the more important the feature that the decision was based on Breiman (2001).

**Figure 2:**
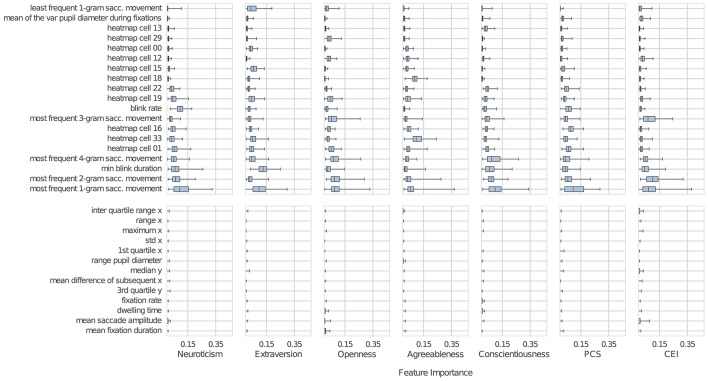
The top half of the figure shows the importance of the top-10 features for each trait, sorted by their median importance across all traits. The bottom half shows the importance of further features that were related to personality or curiosity in prior work. The boxes represent the distribution over feature importance obtained from the 100 models we trained. Each of the boxes spans the inter-quartile range (IQR); the whiskers extend to the minimum and maximum. The dark bar inside each box represents the median. For each classifier, many features remained unused and therefore had an importance of zero. Where most importance values were zero, the box is often invisible.

Figure 2 shows the most important features for our trait-specific classifiers sorted in ascending order by their median importance across all traits. The features were chosen as the smallest set containing the individual ten most important features for each trait according to our method, as well as those features previously linked to personality in Rauthmann et al. (2012), Risko et al. (2012), and Baranes et al. (2015).

As can be seen from Figure 2, five of the 19 most important features are linked to *n*-grams (Bulling et al., 2011), which describe a series of *n* saccades. In contrast to the saccade-based *n*-grams, *n*-grams encoding fixation–saccade sequences are less important. Heatmap features similar to those in Risko et al. (2012), which capture how often a participant looked into certain areas of their visual field, were the second most important class of features. Moreover, the average variance in *pupil diameter during fixations* and *blink rate* turned out to be informative. Complementing the F1 scores that are commonly reported when evaluating machine learning methods with respect to performance, we also provide correlation coefficients between personality scores and the different eye movement features extracted from a sliding window with a length of 15 s (see Table 3 in the Appendix).

## 4. Discussion

One key contribution of our work is to demonstrate, for the first time, that an individual's level of neuroticism, extraversion, agreeableness, conscientiousness, and perceptual curiosity can be predicted only from eye movements recorded during an everyday task. This finding is important for bridging between tightly controlled laboratory studies and the study of natural eye movements in unconstrained real-world environments.

While predictions are not yet accurate enough for practical applications, they are clearly above chance level and outperform several baselines (see Figure 1). The proposed machine learning approach was particularly successful in predicting levels of agreeableness, conscientiousness, extraversion, and perceptual curiosity. It therefore corroborates previous laboratory-based studies that have shown a link between personality traits and eye movement characteristics (Isaacowitz, 2005; Risko et al., 2012; Rauthmann et al., 2012; Baranes et al., 2015).

The trait-specific eye movement characteristics are reliable: Comparing predictions after splitting the recordings into two halves yielded reliability values ranging between 0.63 and 0.83, indicating moderate to strong correlations between predictions derived from the different halves of the recording. The reliability values were lower (0.39–0.63) when the predictions were based on the comparison between two task activities (walking and shopping). These findings suggest that trait-specific eye movements vary substantially across activities. Future work could therefore establish which activities are best suited to elicit trait-specific eye movements, as this could significantly improve both prediction accuracy and reliability for practical applications.

A second contribution of our work is to shed additional light on the close link between personality traits and an individual's eye movements. Thanks to the machine learning approach, we could automatically analyze a large set of eye movement characteristics and rank them by their importance for personality trait prediction. Going beyond characteristics investigated in earlier works, this approach also allowed us to identify new links between previously under-investigated eye movement characteristics and personality traits. This was possible because, unlike classical analysis approaches, the proposed machine learning method does not rely on a priori hypotheses regarding the importance of individual eye movement characteristics. Specifically, characteristics that capture rich temporal information on visual behavior seem to convey fundamental information related to all personality traits, and consistently outperform classic characteristics that have been isolated for investigation in laboratory situations, such as fixation duration (Isaacowitz, 2005; Rauthmann et al., 2012; Risko et al., 2012). By extracting the most important eye movement characteristics for each personality trait (see Figure 2) we also found that the importance of characteristics varies for different personality traits. For example, pupil diameter was important for predicting neuroticism but was less useful for predicting other traits. It is important to note that the goal of the current study was not to shed light on the underlying reasons for why certain eye movement characteristics are more common in particular personality types. Instead, it was specifically designed to explore whether machine learning can be used to classify personality from eye movements in an everyday task.

The prediction accuracy and reliability scores obtained from 42 participants are very promising. However, in computer vision, state-of-the-art machine learning methods are commonly trained on millions of samples (Russakovsky et al., 2015). These large-scale datasets have facilitated data-driven development and automatic learning of features, often outperforming previous manually designed characteristics (Le, 2013). For the field of personality research, obtaining larger datasets with a more representative sample of the general population than the convenience sample of the current study will be an important next step. Consequently, large-scale real-world gaze datasets are likely to improve automatic inference of personality and stimulate research on the automatic representation of gaze characteristics, with the potential to further improve performance as well as deepen our understanding of the interplay between gaze and personality. Importantly, whether the poor performance of our algorithm in predicting openness and CEI is due to the experimental design (relatively small sample and the specific task of running an errand) or due the possibility that there is no link between openness and the way eyes are moved cannot be answered at this stage.

Four important questions arise from our findings: (1) How well do our findings generalize to non-university populations, different personality traits, different settings and other real-world activities? (2) How is the prediction of personality traits affected by temporary user states, such as mood, fatigue or even the person's awareness of the eye tracker (Risko and Kingstone, 2011)? (3) How do gaze-based signals interact with further social cues that are linked to personality, such as body posture (Ball and Breese, 2000) or digital footprints (Youyou et al., 2015)? and (4) how can a system exploit several cues to derive a more holistic view on the user's personality?

Answering these questions will guide research to improve our understanding of how human eye movements are modulated in the real world (Kingstone et al., 2003; Risko and Kingstone, 2011), and how they fit into the broad spectrum of human non-verbal behavior. In turn, improved theoretical understanding will assist the emerging interdisciplinary research field of social signal processing, toward development of systems that can recognize and interpret human social signals (Vinciarelli et al., 2009; Wagner et al., 2011; Vinciarelli and Pentland, 2015).

Such knowledge of human non-verbal behavior might also be transferred to socially interactive robots, designed to exhibit human-like behavior (Fong et al., 2003). These systems might ultimately interact with humans in a more natural and socially acceptable way, thereby becoming more efficient and flexible.

## Author contributions

TL designed and oversaw the study; SM collected the data; SH implemented and evaluated the machine learning method and generated all results and figures; AB advised these analyses; SH, TL, and AB wrote the paper.

### Conflict of interest statement

The authors declare that the research was conducted in the absence of any commercial or financial relationships that could be construed as a potential conflict of interest.

## Footnotes

^1^https://github.molgen.mpg.de/sabrina-hoppe/everyday-eye-movements-predict-personality
