# Supplementary material for: Eye Movements During Everyday Behavior Predict Personality Traits
Source: Front Hum Neurosci. 2018 Apr 13;12:105. doi: 10.3389/fnhum.2018.00105 (PMC5912102; doi:10.3389/fnhum.2018.00105)
Supplement: Supplementary file 1 [file Presentation1.pdf]

Supplementary Material: Eye movements during everyday behavior predict personality traits

Sabrina Hoppe, Tobias Loetscher, Stephanie A. Morey and Andreas Bulling*
*Correspondence:
Andreas Bulling:
bulling@mpi-inf.mpg.de

1 LIST OF EYE MOVEMENT FEATURES

From each time window, we extracted a list of 207 features which are summarised in the list below. For brevity, we sometimes collapsed several items into one bullet point and gave the number of features per bullet points in brackets. E.g. 'mean/variance/minimum/maximum of the fixation duration (4)' means that there are four features, namely statistical mean, variance, minimum and maximum of the fixation duration.

Please note that all code we used to extract these features is publicly available on GitHub[1].

Statistics over fixations, saccades and blinks

Fixations were detected using a dispersion-threshold algorithm with a threshold of 2.5% of the tracking range width (and an additional threshold on the minimum duration of 100ms). This dispersion threshold was also used to define *small* and *large* saccades by comparing the amplitude of a saccade to twice the dispersion threshold.

A saccade that ends right (left) of its point of origin is called *right* (left) saccade. Note that this is independent of the saccade's absolute position, e.g. a saccade within the left lower corner of a person's field of view might still be a right saccade, if it ended a little further right than it started.

A ratio between eye movements is meant to describe the ratio between the number of occurrences in one time window. E.g. if there were 1 fixations and 2 saccade in a time window, the ratio of fixations to saccades would be $\frac{1}{2}$.

The rate of an eye movement is defined as its number of occurrences per second (e.g. 5 fixations per second).

- fixation/saccade rate (2)
- small/large saccade rate (2)
- left/right saccade rate (2)
- saccade to fixation ratio
- ratio of small/large/right/left saccades to the total number of saccades (4)
- mean/variance/minimum/maximum saccade amplitude (4)
- mean/variance/minimum/maximum saccade saccadic peak velocity (4)
- mean/variance/minimum/maximum of the mean pupil diameter during saccades (4)
- mean/variance/minimum/maximum of the fixation duration (4)

[1] https://github.molgen.mpg.de/sabrina-hoppe/everyday-eye-movements-predict-personality

- dwelling time, i.e. sum of all fixation durations
- mean/variance of the mean of angles between subsequent fixations (2)
- mean/variance of the variance of angles between subsequent fixations (2)
- mean of the variance in raw x coordinates during fixations (2)
- mean of the variance in raw y coordinates during fixations (2)
- mean/variance of the mean pupil diameter during fixations (2)
- mean/variance of the variance in pupil diameter during fixations (2)
- mean/variance/minimum/maximum of blink duration (4)
- blinks per second

Statistics over raw gaze data

Features that are computed on the raw **x** coordinates, raw **y** coordinates and pupil **d**iameter separately are abbreviated as on 'XYD'.

- heatmap counts with 8×8 cells (64)
- mean of XYD (3)
- minimum of XYD (3)
- maximum of XYD (3)
- range of XYD (3)
- standard deviation of XYD (3)
- median of XYD (3)
- 1st quartile of the distribution over XYD (3)
- 3rd quartile of the distribution over XYD (3)
- inter quartile range of the distribution over XYD (3)
- absolute value of mean difference between subsequent XYD (3)
- mean difference between subsequent XYD (3)
- mean angle between two subsequent raw gaze points (i.e. between the x-axis and the vector connecting the two gaze points)

Information on the temporal course of saccades and fixations

n-grams describe series of n events, for instance 2-grams based on the events $\{$short fixation, long fixation, saccade$\}$ could be: [short fixation, saccade] or any of the 3^2 possible combinations of events. The frequency of one such n-gram is defined as its number of occurrences here, e.g. the example 2-gram [short fixation, saccade] could occur twice in a given time window. The frequencies for all possible events could be computed, concatenated and then features such as the mean over these frequencies can be extracted.

For n-grams with n from 1 to 4,
each once based on saccades only, once based on saccades and fixations.
For saccades only, each saccade was labelled as small or large, and with one out of 8 possible directions.
For saccades and fixations, a fixation was labelled as either short or long; a saccade was labelled as small or large and with one out of 4 possible directions.

- number of different movements
- highest/lowest frequency within the n-gram (2)
- most/least frequent movements (2)
- range/mean/variance of frequencies within the n-gram (3)

2 PERSONALITY SCORE RANGES

Table S1. Personality score ranges for each personality trait. Participants with raw values smaller or equal to boundary 1 were assigned to personality score range 1, those larger than boundary 1 but smaller or equal to boundary 2 were assigned to score range 2, and those larger than boundary 2 were assigned to score range 3.

trait	boundary 1	boundary 2
Neuroticism	2	2.6
Extraversion	1.9	2.6
Openness	2.5	3
Agreeableness	2.5	3
Conscientiousness	2.1	2.7
PCS	2.5	3
CEI	3	3.6

3 DESCRIPTIVE ANALYSIS

To facilitate comparisons with related work we also provide correlation coefficients between personality scores and those features extracted from a sliding window with a length of 15 seconds (which was the window length that was most frequently selected in our training scheme) in Table S2. These correlation coefficients describe properties of the collected data and, in contrast to the feature importance scores in Figure 2 in the main article, are independent of the machine learning approach.

Table S2. Correlation coefficients between personality score ranges for each trait and features extracted from a sliding window with a length of 15 seconds. The features listed in the table form the smallest set that contains the 15 features with highest correlations for each trait. These 15 coefficients are highlighted in bold. The *n*-gram features are called *sacc.* movements if they were based on saccades only and *SF* if fixations were considered as well.

feature	Neur.	Extr.	Open.	Agree.	Consc.	PCS	CEI
blink rate	**0.39**	-0.20	-0.07	0.03	-0.19	**0.32**	-0.03
heatmap cell 01	-0.12	**0.29**	0.13	-0.27	-0.07	0.16	0.08
heatmap cell 07	-0.12	0.17	**0.17**	0.13	0.13	0.13	0.13
heatmap cell 09	-0.13	0.08	0.11	-0.13	**0.32**	-0.06	-0.05
heatmap cell 10	-0.05	-0.01	0.04	**0.28**	0.20	0.05	0.00
heatmap cell 11	-0.06	0.02	0.01	0.19	-0.04	**0.25**	0.19
heatmap cell 13	0.05	-0.19	0.01	**0.25**	-0.01	-0.01	-0.18
heatmap cell 14	-0.06	0.24	**0.22**	**0.28**	**0.36**	**0.30**	**0.32**
heatmap cell 16	-0.11	**0.29**	0.14	-0.28	-0.06	0.18	0.08
heatmap cell 19	-0.13	**0.30**	0.12	-0.29	-0.04	0.13	0.11
heatmap cell 26	-0.03	**0.28**	**0.24**	**0.31**	**0.43**	**0.23**	**0.27**
heatmap cell 27	-0.14	**0.27**	**0.26**	-0.01	0.26	0.01	0.14
heatmap cell 28	-0.12	0.19	0.16	**0.32**	0.16	0.09	0.12
heatmap cell 29	0.03	**0.33**	**0.22**	**0.28**	0.07	**0.37**	**0.27**
heatmap cell 37	-0.05	0.19	-0.09	0.21	-0.13	**0.31**	**0.25**
heatmap cell 38	0.10	0.00	-0.09	**0.38**	-0.08	0.15	0.05
heatmap cell 45	-0.03	-0.12	0.00	**0.39**	-0.23	**0.23**	0.21
heatmap cell 46	-0.01	0.14	-0.02	**0.36**	-0.01	**0.33**	**0.29**
heatmap cell 47	0.07	**0.36**	-0.01	0.02	0.14	0.13	0.04
heatmap cell 48	**0.25**	0.08	-0.00	-0.23	-0.05	-0.05	-0.22
heatmap cell 49	**0.33**	0.18	0.08	-0.36	-0.13	-0.14	-0.18
heatmap cell 50	**0.22**	-0.16	0.00	0.20	-0.24	-0.01	-0.02
heatmap cell 53	-0.21	-0.04	**0.22**	**0.31**	-0.02	0.07	**0.30**
heatmap cell 54	-0.14	0.13	0.16	**0.32**	0.11	0.15	**0.34**

heatmap cell 55	0.09	**0.33**	0.09	0.07	0.04	0.09	0.10
heatmap cell 56	**0.20**	0.06	0.06	-0.27	-0.08	-0.11	-0.17
heatmap cell 57	**0.25**	0.00	0.15	-0.19	-0.13	-0.02	-0.18
heatmap cell 58	**0.17**	0.05	-0.20	0.23	-0.05	**0.26**	0.12
heatmap cell 59	**0.21**	0.12	-0.09	0.04	-0.07	0.15	-0.02
heatmap cell 61	-0.20	0.05	**0.24**	**0.26**	0.10	0.13	0.15
heatmap cell 62	-0.30	0.11	0.16	0.17	0.25	0.17	**0.37**
heatmap cell 63	0.05	**0.40**	0.12	-0.09	0.06	0.08	0.16
inter quartile range y	0.03	-0.08	-0.12	0.09	-0.12	**0.24**	-0.11
least frequent 1-gram sacc. movement	**0.17**	-0.40	0.17	-0.20	-0.04	-0.18	-0.29
maximumblink duration	-0.26	**0.26**	0.07	0.17	**0.35**	0.12	0.12
maximumfrequency 2-gram sacc. movements	**0.21**	-0.08	-0.01	-0.14	0.01	0.01	-0.08
maximumsaccadic peak velocity	0.07	0.12	-0.21	-0.06	0.26	0.12	**0.25**
maximum x	-0.20	0.08	**0.26**	0.12	0.12	0.16	0.21
mean blink duration	-0.30	**0.30**	0.10	0.12	**0.31**	0.09	0.09
mean diff y	-0.04	-0.20	-0.19	0.01	0.19	**0.23**	-0.07
mean of the var pupil diameter during fixations	-0.08	**0.39**	**0.17**	**0.26**	0.11	**0.37**	**0.42**
mean of the var pupil diameter during saccades	0.10	0.11	-0.10	0.18	0.21	**0.32**	0.17
mean pupil diameter	**0.20**	-0.04	-0.37	**0.25**	-0.08	0.17	0.01
mean saccade amplitude	0.04	-0.22	-0.17	-0.36	**0.27**	-0.12	-0.22
mean saccadic peak velocity	0.01	0.13	-0.18	-0.01	**0.41**	0.10	0.19
min blink duration	-0.27	**0.28**	0.10	0.05	0.21	0.07	0.02
min fixation duration	-0.03	0.01	**0.32**	0.09	-0.24	0.07	-0.21
minimum pupil diameter	-0.23	0.09	**0.17**	0.20	0.25	-0.06	-0.09
minimum x	**0.19**	0.05	-0.18	-0.06	0.05	-0.06	-0.05
most frequent 2-gram SF movement	0.05	-0.03	-0.19	-0.22	**0.31**	0.04	0.02
most frequent 4-gram sacc. movement	-0.05	-0.17	0.02	-0.15	**0.42**	0.08	0.10
range of frequencies of 2-gram sacc. movements	**0.19**	-0.10	0.08	-0.05	-0.10	-0.07	-0.19
range of frequencies of 3-gram sacc. movements	0.02	0.13	**0.18**	-0.01	-0.00	-0.01	0.01
ratio of large saccades	0.00	-0.18	-0.23	-0.32	**0.29**	-0.04	-0.08
ratio of right saccades	0.00	0.08	-0.27	-0.04	**0.38**	0.13	0.08
ratio of small saccades	0.13	**0.37**	**0.21**	0.20	-0.01	**0.32**	**0.32**
small saccade rate	**0.17**	0.19	0.04	0.01	-0.17	0.17	**0.24**
var blink duration	-0.29	0.18	-0.14	0.23	**0.37**	0.22	0.18
var frequency of 1-gram sacc. movements	**0.18**	-0.01	-0.08	-0.15	-0.13	-0.03	-0.13
var of mean pupil diameter during fixations	-0.12	0.25	**0.21**	**0.28**	**0.55**	0.14	**0.23**
var of the mean pupil diameter during saccades	0.00	0.20	0.12	0.21	**0.55**	0.22	0.17
var of the var pupil diameter during fixations	-0.16	**0.31**	**0.22**	0.18	0.01	**0.31**	**0.31**
var saccadic peak velocity	0.04	0.19	-0.14	0.03	**0.37**	0.11	**0.27**

4 TRAINING PROCEDURE

In the following, the scheme to train a single random forest classifier will be described (see GitHub for our implementation):
A priori, no clear hypotheses about useful window sizes for the sliding window approach or informative features for every day life recordings existed. Therefore, each classifier was trained in a *nested cross validation* scheme that fits parameters during training while preventing overfitting. Cross validation means that a given set of recordings is not simply split into one training and one test set, but instead the algorithm cycles different splits into the training and test sets. On each pair of training and test set, a separate classifier is trained and the performance measure is then averaged over all splits. Typically, a single classifier is trained on the training set and is later evaluated on the unseen test set. In *nested* cross validation however, another (so-called inner) cross validation takes place on the training set. This is, the algorithm again cycles through different splits of the (outer) training set into (inner) training and (inner) test set. In this inner cross validation, different parameters can be tested on the inner training set and applied to the inner test set. Once the best performing parameters (i.e. in our case window size and features) are chosen, a classifier with these parameters is newly trained on the entire outer training set and evaluated on the still unseen outer test set. Figure S1 shows the distribution of all chosen window sizes.

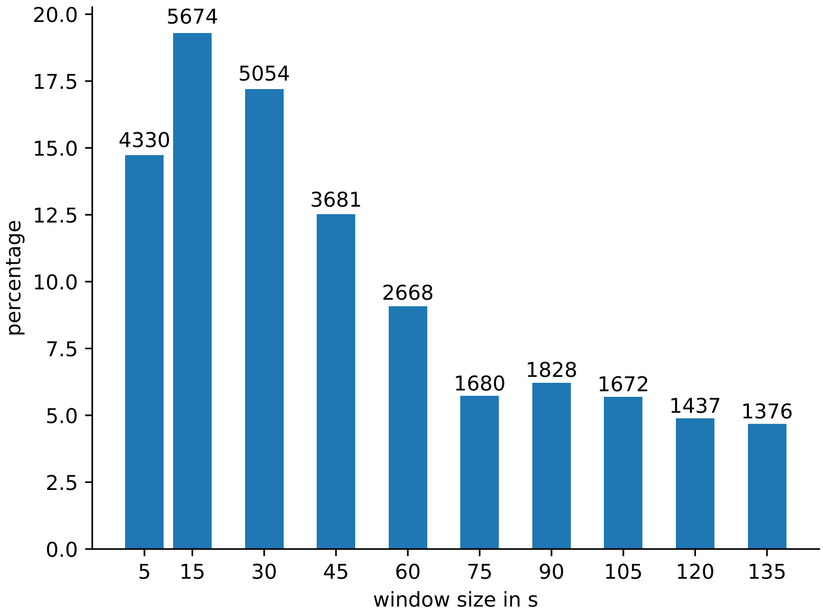

Figure S1. During the training procedure, a nested cross validation scheme is used to determine the best performing parameters, one of which is the size of a sliding window used to process raw gaze data. This histogram shows the frequency of each window size (x-axis) summed up over all cycles of the training scheme. While the y-axis shows relative frequency, the number above each bar is the absolute number of times a window was chosen.

The inner cross validation cycle included 3 different data splits (folds) that were chosen to contain an approximately equal number of samples for each class. The goodness of a classifier on the inner test set was evaluated using the accuracy over all samples (i.e. independent of the participant they belong to). For the outer cross validation, the data used for testing is cycled such that each time, 5 different participants are in the test set and the remaining 37 in the training set. Finally, a prediction for each participant in the outer

test set was derived by majority voting over all time windows associated with that particular participant. The predictions from all outer folds were merged into a set of exactly one prediction per participant and evaluated in terms of an F1 score with macro averaging. The F1 score is defined as the harmonic mean of precision and recall, where precision for a certain class captures how many of the actual class instances were correctly detected, whereas recall captures how many of the instances that were predicted to be class instances were actually correct. Macro averaging means that one F1 score per class is computed and those are then averaged. This reflects the intuition that a personality predictions system is expected to predict extreme values, as opposed to a system that typically predicts the most frequent personality class.
Feature selection within the inner cross validation cycle is performed based on the Random Forests' feature importance: all features that did not reach a higher average importance than 0.005 across all splits were disabled for the outer cross validation.
